# The Power of Intervention: Tackling Alzheimer’s and Mental Health Disparities in Aging Populations

**DOI:** 10.1093/geroni/igaf122.1528

**Published:** 2025-12-31

**Authors:** María Aranda

**Affiliations:** University of Southern California, Los Angeles, California, United States

## Abstract

María P. Aranda, PhD, MSW, MPA, LCSW is an internationally-recognized social worker and sociobehavioral scholar in the fields of social work, gerontology, and health equity. She is Professor of Social Work with a joint appointment in Gerontology at the University of Southern California, and holds the Margaret W. Driscoll/Louise M. Clevenger Professorship in Social Policy and Administration. Dr. Aranda specializes in the area of medical and psychiatric comorbidity in adult and older adult populations specifically in the areas of Alzheimer’s disease, depression, psychosocial interventions for individuals and family care partners, health disparities and ADRD. Dr. Aranda is the Executive Director of the USC Edward R. Roybal Institute on Aging. She has extensive experience in randomized trials, ethnographic methods, large-scale epidemiological research, stakeholder engagement, and innovative recruitment and retention protocols with underrepresented racial and ethnic groups in the US and international populations.

